# Gemcitabine and Nucleos(t)ide Synthesis Inhibitors Are Broad-Spectrum Antiviral Drugs that Activate Innate Immunity

**DOI:** 10.3390/v10040211

**Published:** 2018-04-20

**Authors:** Hye Jin Shin, Chonsaeng Kim, Sungchan Cho

**Affiliations:** 1 Center for Convergent Research of Emerging Virus Infection, Korea Research Institute of Chemical Technology, 141 Gajeong-ro, Yuseong-gu, Daejeon-si 34114, Korea; shinhy@krict.re.kr; 2 Anticancer Agent Research Center, Korea Research Institute of Bioscience & Biotechnology, 30 Yeongudanji-ro, Ochang-eup, Cheongwon-gu, Cheongju-si, Chungbuk 28116, Korea; 3 Department of Biomolecular Science, KRIBB School of Bioscience, Korea University of Science and Technology, 217 Gajeong-ro, Yuseong-gu, Daejeon-si 34113, Korea

**Keywords:** nucleoside analog, gemcitabine, antiviral drugs, innate immunity, interferon-stimulated gene, nucleos(t)ide synthesis

## Abstract

Nucleoside analogs have been frequently identified as antiviral agents. In recent years, gemcitabine, a cytidine analog in clinical use for the treatment of many solid tumors, was also shown to have antiviral activity against a broad range of viruses. Nucleoside analogs generally interfere with cellular nucleos(t)ide synthesis pathways, resulting in the depletion or imbalance of (d)NTP pools. Intriguingly, a few recent reports have shown that some nucleoside analogs, including gemcitabine, activated innate immunity, inducing the expression of interferon-stimulated genes, through nucleos(t)ide synthesis inhibition. The precise crosstalk between these two independent processes remains to be determined. Nonetheless, we summarize the current knowledge of nucleos(t)ide synthesis inhibition-related innate immunity and propose it as a newly emerging antiviral mechanism of nucleoside analogs.

## 1. Introduction

Nucleoside analogs have been historically used for anti-cancer chemotherapy because they inhibit cellular DNA/RNA polymerases [1]. More recently, nucleoside analogs have expanded their therapeutic applications and are being used to develop antiviral drugs against a wide range of serious and life-threatening viruses. Some nucleoside analog drugs targeting specific viral polymerases (acyclovir for herpesviruses, zidovudine for human immunodeficiency virus (HIV), and sofosbuvir for hepatitis C virus (HCV)) have been successful in clinical trials [2,3,4,5] and are currently in use for the treatment of virus-infected patients. Another class of nucleoside analog drugs such as ribavirin, more broadly-acting on various viruses, has been used in conjunction with IFN-α [6]. Importantly, extensive studies on the antiviral action of ribavirin have established the underlying molecular framework of nucleoside analogs.

**Figure 1:**
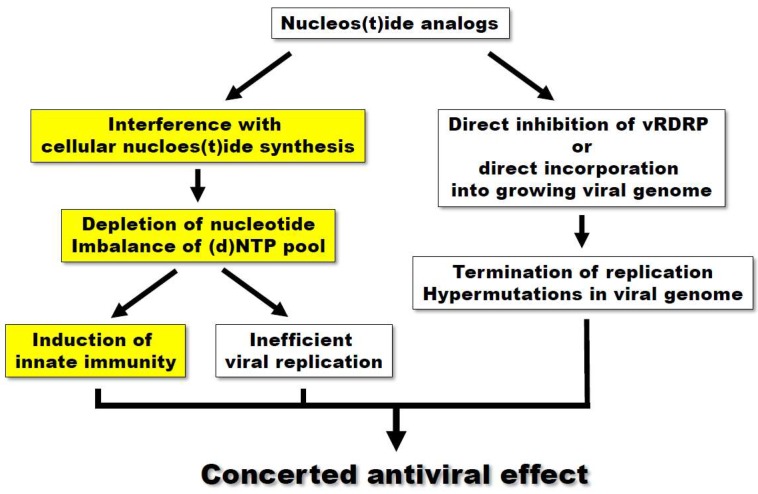
The mechanism of antiviral effect of nucleos(t)ide analogs. Nucleos(t)ide synthesis inhibition-related innate immunity, a newly emerging antiviral mechanism of nucleoside analogs, was highlighted by yellow boxes.

The primary mechanism to explain the antiviral effect of nucleoside analogs is based on their direct action on viral polymerization. Nucleoside analogs are transported into the cells and phosphorylated by the consecutive action of viral or cellular kinases, eventually generating nucleotide triphosphates. Mature nucleotide analogs, which are similar to physiological nucleotides, can directly incorporate into the growing viral genome during polymerization, resulting in the termination of chain reaction or the accumulation of mutations (Figure 1). Alternatively, nucleotide analogs can bind to the nucleotide-binding region on viral polymerases and block the entry of incoming natural nucleotides. The other mechanism is based on the modulation of cellular nucleos(t)ide synthesis. There have been accumulating reports that nucleoside analogs act as antiviral agents by interfering with host nucleos(t)ide synthesis pathways [7,8,9,10]. By targeting metabolic enzymes(s), nucleoside analogs block the natural flow of nucleos(t)ide synthesis and consequently cause the depletion or imbalance of (d)NTP pools. As viral replication is highly dependent on the availability of host nucleotides, a nucleotide-defective condition decreases the efficiency of viral replication. A more recently proposed mechanism has been based on the observations that a few nucleoside analogs activate innate immunity, especially involving the upregulation of interferon-stimulated genes (ISGs). Importantly, this phenomenon is usually mediated by the inhibition of nucleotide synthesis, suggesting a potential crosstalk between nucleotide biosynthesis and innate immunity. However, the precise mechanism of this crosstalk remains to be elucidated.

There is now an increasing number of nucleoside analogs with antiviral activity toward a wide range of viruses. They have been well-summarized in a previous report [1]. In the present review, we focus more on gemcitabine as a nucleoside analog, which is clinically relevant and whose broad-spectrum antiviral activity has been recently reported by many groups including our group. More importantly, we summarize inhibitors of the purine/pyrimidine biosynthesis pathways that induce innate immunity and propose possible mechanisms of action for these inhibitors.

## 2. The Broad-Spectrum Antiviral Activity of Gemcitabine

Gemcitabine is a cytidine analog that has been clinically used for the treatment of various cancers [11,12]. However, in recent years, the antiviral activity of gemcitabine has also been reported against a broad range of RNA viruses, including Middle East respiratory syndrome coronavirus (MERS-CoV), severe acute respiratory syndrome coronavirus (SARS-CoV), Zika virus (ZIKV), HCV, poliovirus (PV), influenza A virus (IAV), HIV, and enteroviruses (EV) [13,14,15,16,17,18].

The antiviral activities of gemcitabine against the abovementioned viruses are summarized in Table 1. MERS-CoV and SARS-CoV belong to the family of Coronaviridae and are causative agents of severe viral respiratory illness in humans. To efficiently select appropriate antiviral drug candidates, Dyall et al. screened 290 FDA-approved drugs in virus-infected Vero E6 cells and identified gemcitabine as one of drugs with antiviral activity against both MERS-CoV and SARS-CoV (EC_50_ of 1.2 μM and 4.9 μM, respectively) [13]. More recently, gemcitabine was shown to effectively suppress ZIKV infection and replication in human retinal pigment epithelium (RPE) cells, particularly at non-cytotoxic concentrations (EC_50_ of 0.01 μM vs. CC_50_ of > 10 μM) [14]. ZIKV, a member of the Flaviviridae family, can infect pregnant women and cause congenital abnormalities such as microcephaly in infants, which has attracted increasing public attention as well as extensive research and development into possible treatments. Effective antiviral activities of gemcitabine were also found for the replication of HCV in Huh-7 cells and the infection of HIV in U373-MAGI-CXCR4_CEM_ cells, with estimated EC_50_s of 12 nM and 16.3 nM, respectively [17,19], which were lower concentrations than those used in cancer therapy [20]. In the case of HIV, the combination of gemcitabine with decitabine, another nucleoside analog in clinical use for cancer therapy, synergistically reduced HIV infectivity by increasing the viral mutation frequency [21]. In a follow up study, Clouser et al. further reported the antiviral effect of gemcitabine against HIV-related retrovirus, murine leukemia virus (MuLV), in vitro (EC_50_ of 1.6 nM) and even in murine AIDS model [17]. A significant antiviral effect of gemcitabine on IAVs was also reported for RPE cells by Denisova et al. (EC_50_ of 0.068 μM) [16]. They also tested whether gemcitabine had an antiviral effect on several other viruses of different families and found its strong inhibitory effect on Sindbis virus and herpes simplex virus-1 (HSV-1) (>2 log reduction in virus titer) but relatively weak effects on Semliki forest virus and human echovirus 6, and minimal effects on Bunyamwera virus, measles virus (MeV), and vaccinia virus [16]. The antiviral effect of gemcitabine on EVs, initially performed on Coxsackievirus B3 (CVB3), was found from screening FDA-approved drugs in CVB3 replicon-harboring Vero cells by our group (EC_50_ of 0.4 μM) [18]. Its broad-spectrum antiviral activity on EVs was further identified by observing a similar inhibitory effect on enterovirus 71 (EV71) and human rhinoviruses (HRVs) (EC_50_s of 1 and 1–5 μM, respectively). In the case of HRV, the antiviral effect of gemcitabine was further confirmed in a virus-infected mouse model [22]. In this study, intranasal administration of gemcitabine significantly lowered the pulmonary viral load and inflammation by decreasing proinflammatory cytokines, including TNF-α and IL-1β, and the number of lung infiltrating lymphocytes. More recently, Zhang et al. also identified gemcitabine as the best anti-PV inhibitor from a screen of FDA-approved drugs in PV replicon-harboring HeLa cells (EC_50_ of 0.3 µM) [15].

As previously mentioned, accumulating evidence has definitively demonstrated that gemcitabine is an effective broad-spectrum inhibitor of RNA viruses and has a therapeutic potential for the treatment of various virus-associated diseases. Moreover, it is possible that gemcitabine is effective for other untested RNA viruses. Because gemcitabine is a deoxycytidine analog that interferes with DNA as well as RNA synthesis, DNA viruses may not be the exception. Consistent with this possibility, there has been a report that the infection of HSV-1, which is a representative DNA virus classified into the Herpesviridae family, was strongly affected by gemcitabine [16]. Most of the abovementioned viruses have, at best, limited prophylactic or therapeutic drugs as possible treatments. This is especially true for newly emerging or re-emerged viruses involving serious illnesses, such as MERS-CoV, SARS-CoV, and ZIKV, which are major threats to public health and which urgently need an effective treatment during their early stages of infection. In this regard, repurposing of gemcitabine for the treatment of patients infected with these deadly viruses is a realistic approach. Importantly, it is noteworthy that ZIKV was the most strongly affected by gemcitabine, with a low nanomolar EC_50_, which was lower than that used in cancer therapy [14,20]. Even for other viruses with a relatively high EC_50_, there is an option to treat patients with a combination of gemcitabine with other antiviral agents. In this manner, an effective antiviral treatment may be achieved by the synergistic action of two antivirals with much lower doses for each drug, which minimizes deleterious side effects when used clinically. As an example, the synergistic antiviral effect of gemcitabine in combination with ribavirin, an antiviral drug currently being used against a few RNA viruses, was reported against EVs such as CVB3 and EV71 [18]. As previously mentioned, the combination of gemcitabine with decitabine synergistically suppressed HIV infectivity both in vitro and in vivo [17,21]. However, the actual use of gemcitabine in virus-infected patients necessitates prior in vivo animal studies and clinical trials. Even though most antiviral data have originated from in vitro studies, two recent studies have reported the antiviral effects of gemcitabine in murine models [17,22]. More extensive analyses of gemcitabine in animal models in the near future will accelerate its therapeutic applications in clinical trials.

## 3. Innate Immune Activation by Purine and Pyrimidine Biosynthesis Inhibitors

Most studies regarding the antiviral activity of gemcitabine lack experimental evidence of the mode of action. However, our group has recently reported that gemcitabine had an anti-EV effect by targeting the salvage pathway of pyrimidine biosynthesis [23]. Moreover, gemcitabine strongly induced the expression of several ISGs including CXCL10, IRF7, IRF9, IFIT1, and DDX58, which were the major effectors in the innate immunity that defended the host against the virus infection. These results were consistent with a previous report that gemcitabine stimulated the production of IFN-β and IFN-γ in IAV-infected RPE cells [16]. Importantly, the activation of ISGs was well-correlated with the inhibition of pyrimidine biosynthesis, suggesting a link between pyrimidine biosynthesis and innate immunity. Similar phenomena in terms of ISG activation have been previously reported with a few compounds out of several purine or pyrimidine biosynthesis inhibitors that had antiviral activity, as summarized in Table 2 [6,10,23,24,25,26,27,28,29,30,31,32,33,34,35,36,37,38,39,40,41,42].

Regarding purine biosynthesis inhibitors, ribavirin and mycophenolic acid (MPA) are inhibitors of inosine-5′-monophosphate (IMP) dehydrogenase (IMPDH), which is a key enzyme of the purine biosynthesis pathway. These inhibitors have been successfully used as clinical antiviral or immunosuppressant agents for decades. Both have antiviral activities against viruses such as HCV, hepatitis E virus (HEV), MERS-CoV, dengue virus, yellow fever, hepatitis B virus, West Nile virus (WNV), Chikungunya virus (CHIKV), and IAV [24,25,26,27,28,29,30], majorly through the inhibition of the purine biosynthesis pathway, with the antiviral activity against HCV and HEV shown to involve the stimulation of ISGs [10,30]. For the antiviral activity of ribavirin against HCV, ribavirin specifically induced the expression of IRF7, IRF9, and ISG15 mRNAs, which are known to be important for anti-HCV immune responses [6]. ISG activation occurred through an undefined mechanism that was different from the classical IFN signaling, intracellular dsRNA sensing pathway, Toll-like receptor and nuclear factor B pathways. More importantly, ribavirin-induced ISG activation and antiviral activity were suppressed using supplemented guanosine, a natural analog of ribavirin, suggesting IMPDH inhibition-mediated ISG activation as an alternative innate immunity pathway. Like ribavirin, MPA remarkably induced the expression of several ISGs, including IRF1, IRF9, ISG15, IFI6, IRF7, CXCL10, IFIT2, and IFITM3 mRNAs in naïve or HEV-infected Huh-7 cells, and the induction of ISGs was at least partially abrogated by the use of supplemented guanosine [10]. Mechanistically, the induction of ISGs by MPA was independent of the classical JAK/STAT system, which is similar to that observed with ribavirin [30]. Similar results were obtained with several IMPDH1 or IMPDH2 inhibitors, with various affinities, that were custom-designed and synthesized [10].

As shown in Table 2, most pyrimidine biosynthesis inhibitors target dihydroorotate dehydrogenase (DHODH), an essential enzyme in de novo pyrimidine synthesis. Lucas-Hourani et al. identified DD264 as an interferon-sensitive response element (ISRE)-stimulating compound from high-throughput screening, and further analyses suggested that it was a DHODH inhibitor with a strong antiviral activity against various viruses including MeV, CHIKV, and WNV [37]. DD264 enhanced the expression of several ISGs, which were almost completely suppressed by the addition of supplemented uridine, indicating DHODH inhibition-mediated ISG activation. Moreover, the antiviral activity of and ISG activation by DD264 required the interferon regulatory factor 1 (IRF1) transcription factor, a master regulator of antiviral gene expression [37], which was consistent with the observation that the anti-HCV activity of MPA was partially mediated by IRF1 [30]. In this study, similar results were shown with brequinar, another well-known DHODH inhibitor. FA-613 is also an antiviral compound, which inhibits the pyrimidine biosynthesis pathway, probably via targeting DHODH and inducing the expression of ISGs such as IFNB1, CXCL10, ISG15, and CCL5 [38]. However, whether ISG activation is mediated by pyrimidine biosynthesis inhibition remains to be determined.

The mechanism of nucleotide synthesis inhibitor-induced ISG activation is still presently unclear. Nevertheless, there has been accumulating evidence showing that nucleotide synthesis inhibitor-induced ISG activation is independent of the classical JAK/STAT-mediated IFN signal [6,10,23]. First, Wang et al. clearly showed that ISG activation and anti-HEV activity induced by MPA or brequinar was not mediated by JAK [10]. Second, IRF7 induction by ribavirin was not affected by knockdown of STAT1, while that of IFN-α was strongly affected under the same conditions [6]. Third, our recent study with gemcitabine further confirmed IFN signal-independent ISG activation by parallel studies comparing the effects of gemcitabine and IFN-α. In our study, the phosphorylation of STAT1 at Tyr701, which was dramatically triggered by IFN-α, did not occur when treated with gemcitabine [23]. Moreover, the upregulation of DDX58 mRNAs induced by gemcitabine was not affected by IRF9 knockdown, which was contrary to the result that IFN-α-induced upregulation of DDX58 mRNAs was significantly suppressed under the same conditions. Consistent with above observations, there have been some reports that ISGs was induced in the absence of JAK1 or STAT1 activation [43,44].

Despite limited data, we speculate the scenario of ISG activation that is independent of JAK/STAT-mediated IFN signal. Purine or pyrimidine biosynthesis inhibitors could interfere with the metabolic pathway through targeting some key enzymes such as IMPDH and DHODH, leading to the depletion or imbalance of the (d)NTP pool. Inactivation of metabolic enzyme(s) itself or consequently altered nucleos(t)ide pools might trigger a signal, which is ultimately delivered to certain *cis*-acting elements on the promoter of a subset of ISGs, possibly through the relay of kinases and transcription factors. Based on the previously mentioned reports, this signal is less likely to be dependent on STAT1/2-IRF9 (IFN-stimulated gene factor 3; ISGF3), at least for gemcitabine, which is the major transcriptional complex in the IFN-induced JAK/STAT pathway. It should also be considered that Thomas et al. excluded the involvement of an intracellular double-stranded RNA sensing pathway, Toll-like receptor and nuclear factor κB pathways, as well as a classical IFN signal in the activation of ISGs induced by ribavirin [6]. Despite the consensus of ISG activation, each purine/pyrimidine biosynthesis inhibitor seems to induce distinct sets of ISGs, at least with different patterns [10]. Targeting an enzyme in which pathways (purine or pyrimidine synthesis) or steps (early/late and de novo/salvage) produce different levels of intermediates and nucleos(t)ides will consequently result in diverse outcomes of ISG activations. There might be more than one signaling pathway involved. The synergistic antiviral activity of gemcitabine and ribavirin observed in our study might be explained by the possible existence of two separate signaling pathways that mediate each inhibition of nucleotide synthesis toward ISG activation. Systematic analyses of signaling kinases, IRFs, and STATs using siRNA knockdown and/or pharmacological inhibition and metabolic analyses of corresponding intermediates and nucleos(t)ides should therefore clarify the underlying molecular mechanisms of ISG activation by purine/pyrimidine biosynthesis inhibitors.

## 4. Conclusions

As newly emerging or re-emerged viruses such as SARS-CoV, MERS-CoV, and ZIKV have become a major threat to public health, the need for broad-spectrum antiviral drug has increased. In this regard, nucleoside analogs that directly target viral RNA-dependent RNA polymerase and present a high barrier to the development of resistant viruses have been considered advantageous. Moreover, recent discovery of a new antiviral mode of nucleoside analogs acting through innate immunity strengthens the molecular basis for their therapeutic application as broad-spectrum antiviral drugs.

Nucleoside analogs probably induce different subsets of ISGs, at least with a different pattern, leading to various combinations of ISGs and resulting antiviral outcomes. Moreover, according to Schoggins et al., different viruses are affected by distinct subsets of ISGs and some ISGs such as IRF1, MB21D1, HPSE, DDX58, MDA, and IFITM3 act broadly on various viruses [45]. Thus, more systematic analyses on the subsets of ISGs induced by antiviral nucleoside analogs are required for the identification of better antiviral drugs that can be used broadly or specifically. Given the clinical side effects of IFN treatment, nucleotide analogs that differ from IFN in the activation of subsets of ISGs need to be considered as alternatives. Nevertheless, nucleoside analogs interfering with the host nucleotide synthesis pathway suggest possible side effects in their clinical applications. Careful evaluation of clinical safety is required and their application for the urgent measure of patients infected with deadly viruses would be worth being primarily considered.

## Figures and Tables

**Table 1:** Broad-spectrum antiviral activity of gemcitabine.

Virus	Group	Family	Inhibition of	Cell Line/Animal Model	Antiviral Activity (EC_50_ or IC_50_)	Cell Toxicity (CC50)	Ref.
MERS-CoV	(+)ssRNA	Coronaviridae	Viral replication	Vero E6 cells	1.2 μM	>10 μM	[13]
SARS-CoV	(+)ssRNA	Coronaviridae	Viral replication	Vero E6 cells	4.9 μM	>10 μM	[13]
ZIKA	(+)ssRNA	Flaviviridae	Viral RNA and protein synthesis virus-mediated cell death	RPE cells	0.01 μM	>10 μM	[14]
HCV	(+)ssRNA	Flaviviridae	Viral replication	Huh-7 cells	12 nM	>44 μM	[19]
Poliovirus	(+)ssRNA	Picornaviridae	Viral replication RNA polymerase	HeLa cells	0.3 μM	>100 μM	[15]
Influenza A virus (IAV)	(−)ssRNA	Orthomyxoviridae	Viral RNA transcription and replication virus entry	RPE cells	0.068 μM	>10 μM	[16]
HIV	ssRNA-RT	Retroviridae	Viral replication	U373-MAGI-CXCR4_CEM_ cells	16.3 nM		[17]
MuLV	ssRNA-RT	Retroviridae	Viral replication	U373-MAGI-CXCR4_CEM_ ls/Murine AIDS mouse model	1.6 nM		[17]
CVB3	(+)ssRNA	Picornaviridae	Viral proliferation Viral replication Early step of virus infection	Vero cells HeLa cells	0.4 μM 2 μM	>50 μM >50 μM	[18]
EV71	(+)ssRNA	Picornaviridae	Viral proliferation Viral replication	Vero cells	1 μM	>50 μM	[18]
HRV	(+)ssRNA	Picornaviridae	Viral RNA synthesis	HeLa cells/Mouse	1–5 μM	>50 μM	[18,22]
Sindbis virus (SINV)	(+)ssRNA	Togaviridae	Virus production	Vero cells	>2 logs (at 3 μM)		[16]
HSV-1	dsDNA	Herpesviridae	Virus production	RPE cells	>4 logs (at 3 μM)		[16]

RPE cells: retinal pigment epithelium cells; U373-MAGI-CXCR4CEM cells: cells expressing the CD4 receptor and the CXCR4 coreceptor.

**Table 2:** Purine & Pyrimidine biosynthesis inhibitors.

Purine					Pyrimidine				
**Compound**	**Target**	**Antiviral Ref.**	**Innate Immunity Ref.**	**Chemical Structure (Pubchem)**	**Compound**	**Target**	**Antiviral Ref.**	**Innate Immunity Ref.**	**Chemical Structure (Pubchem)**
**Mycophenolic acid**	IMPDH	[24,25,26,27,28,29]	[29,30]	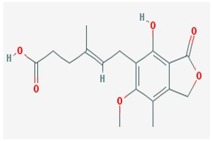	**Gemcitabine**	Cytosine→Cytidine Uracil→Uridine	Table 1	[23]	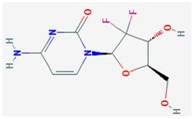
**Tiazofurin**	IMPDH	[31]		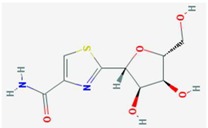	**Brequinar**	DHODH	[34]	[10]	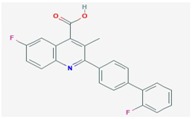
**Mizoribine**	IMPDH, GMP synthetase	[32]		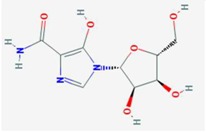	**Leflunomide** **Teriflunomide**	DHODH	[35,36]		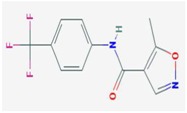
**Ribavirin**	IMPDH	[6,26,27,28]	[6]	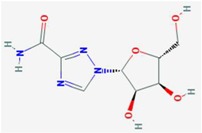	**6-Azauridine**	ODCase	[10,25,26]	[10]	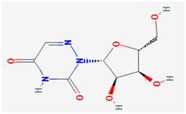
**Favipiravir (T-705)**	?	[33]		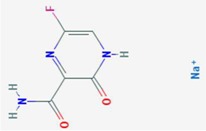	**DD264**	DHODH	[37]	[37]	
					**FA-613**	DHODH	[38]	[38]	
**1346, 1347, 1348**	IMPDH	[10]	[10]		**A3**	DHODH	[39]		
					**6b/14b**	DHODH/CAD	[40]		
					**NITD-982**	DHODH	[41]		
					**D282**	DHODH	[42]		
